# Supplementary material for: Inflaming Public Interest: A Qualitative Study of Adult Learners’ Perceptions on Nutrition and Inflammation
Source: Nutrients. 2020 Jan 28;12(2):345. doi: 10.3390/nu12020345 (PMC7071230; doi:10.3390/nu12020345)
Supplement: Supplementary file 1 [file nutrients-12-00345-s001.pdf]

Table S1. Qualitative Research Review Guidelines (RATS) checklist.

Qualitative Research Review Guidelines – RATS [1]		
**Ask this of the manuscript**	**This should be included in the manuscript**	**Where item has been addressed**
**R – Relevance of study design**		
Is research question interesting?<br><br>Is research question relevant to clinical practice, public health, or policy?	Research question explicitly stated<br><br>Research question justified and linked to the existing knowledge base (empirical research, theory, policy)	Question provided on page 1 and objectives provided on pages 2 and 6<br><br>Pages 2 through to 6
**A – Appropriateness of qualitative method**		
Is qualitative methodology the best approach for the study aims?<br>*Interviews*: experience, perceptions, behavior, practice, process<br>*Focus groups*: group dynamics, convenience, non-sensitive topics<br>*Ethnography*: culture, organizational behavior, interaction<br>*Textual analysis*: documents, art, representations, conversations	Study design described and justified e.g., why was a particular method (i.e., interviews) chosen?	Pages 6 through to 8
**T – transparency of procedures**		
Sampling<br>Are the participants selected the most appropriate to provide access to the type of knowledge sought by the study?<br><br>Is the sampling strategy appropriate?	Criteria for selecting the study sample justified and explained<br>*theoretical*: based on pre conceived or emergent theory<br>*purposive*: diversity of opinion<br>*volunteer*: feasibility, hard-to-reach groups	Page 8
Recruitment<br>Was recruitment conducted using appropriate methods?<br><br>Is the sampling strategy appropriate?<br><br>Could there be selection bias?	Details of how recruitment was conducted and by whom<br><br>Details of who chose not to participate and why	Pages 6 through to 8<br><br>Pages 6 through to 8

Data collection<br>Was collection of data systematic and comprehensive?<br><br>Are characteristics of study group and setting clear?<br><br>Why and when was data collection stopped, and is this reasonable?	Method (s) outlined and examples given (e.g., interview questions)<br><br>Study group and setting dearly described<br><br>End of data collection justified and described	Page 7, Appendix Figure 1A and Appendix Table 2A<br><br>Page 6 and 7<br><br>Page 7
Role of researchers<br>Is the researcher(s) appropriate?<br><br>How might they bias (good and bad) the conduct of the study and results?	Do the researchers occupy dual roles (clinician and researcher)?<br><br>Are the ethics of this discussed? Do the researcher(s) critically examine their own influence on the formulation of the research question, data collection, and interpretation?	Page 10 and 11<br><br>Page 23
Ethics<br>Was informed consent sought and granted?<br><br>Were participants'' anonymity and confidentiality ensured?<br><br>Was approval from an appropriate ethics committee received?	Informed consent process explicitly and dearly detailed<br><br>Anonymity and confidentiality discussed<br><br>Ethics approval cited	Page 8<br><br>Page 8<br><br>Page 8
**S - Soundness of interpretive approach**<br><br>*Analysis*<br>Is the type of analysis appropriate for the type of study?<br>*Thematic*: exploratory, descriptive, hypothesis generating<br>*Framework:* e.g. policy<br>*Constant comparison/grounded theory:* theory generating, analytical<br><br>Are the interpretations dearly presented and adequately supported by the evidence?<br><br>Are quotes used and are these appropriate and effective?	 <br><br>Analytic approach described in depth and justified<br><br>Indicators of quality:<br>Description of how themes were developed from the data (inductive or deductive)<br>Evidence of alternative explanations being sought<br>Analysis and presentation of negative or deviant cases<br>Description of the basis on which quotes were chosen<br>Semi-quantification when appropriate<br>Illumination of context and/or meaning, richly detailed	 <br><br>Pages 8 through to 10<br><br>Pages 8 through to 10, Appendix Table 3A, 4A and 5A<br><br><br><br><br><br>Table 3 and Appendix Table 5A

Was trustworthiness/reliability of the data and interpretations checked?	Method of reliability check described and justified<br>e.g., was an audit trail, triangulation, or<br>member checking employed?<br><br>Did an independent analyst review data and contest themes? How were disagreements resolved?	Pages 8 through to 10 and Appendix Table 4A
Discussion and presentation<br>Are findings sufficiently grounded in a theoretical or conceptual framework?<br><br>Is adequate account taken of previous knowledge and how the findings add?<br><br>Are the limitations thoughtfully considered?<br><br>Is the manuscript well written and accessible?	Findings presented with reference to existing theoretical and empirical literature, and how they contribute<br><br>Strengths and limitations explicitly described and discussed<br><br>Evidence following guidelines (format, word count)<br><br>Detail of methods or additional quotes, contained in appendix<br><br>Written for a health sciences audience	Pages 18 through to 22<br><br>Pages 22 and 23<br><br>Pages 1 through to 29 and Title page<br><br>Appendix
Are red flags present?<br><br>These are common features of ill-conceived or poorly executed qualitative studies, are a cause for concern, and must be viewed critically<br><br>They might be fatal flaws, or they may result from lack of detail or clarity	Grounded theory; not a simple content analysis but a complex, sociological, theory generating approach<br><br>*Jargon*: descriptions that are trite or jargon filled should be viewed skeptically<br><br>*Over interpretation*: interpretation must be grounded in "accounts" and semi-quantified if possible or appropriate<br><br>*Seems anecdotal, self-evident*: may be a superficial analysis, not rooted in conceptual framework or linked to previous knowledge, and lacking depth	-

	Consent process thinly discussed: may not have met ethics requirements<br><br>*Doctor-researcher*: consider the ethical implications for patients and the bias in data collection and interpretation	

[1] The RATS guidelines modified for BioMed Central are copyright Jocalyn Clark, BMJ. They can be found in Clark JP: **How to peer review a qualitative manuscript**. In *Peer Review Health Sciences*. Second edition. Edited by Godlee F, Jefferson T. London: BMJ Books; 2003:219-235
